# Distinct roles of MK2 and MK5 in cAMP/PKA- and stress/p38^MAPK^-induced heat shock protein 27 phosphorylation

**DOI:** 10.1186/1750-2187-6-4

**Published:** 2011-05-16

**Authors:** Alexey Shiryaev, Gianina Dumitriu, Ugo Moens

**Affiliations:** 1 University of Tromsø, Faculty of Health Sciences, Department of Medical Biology, Host-Microbe Interaction Research Group, N-9037 Tromsø, Norway

## Abstract

**Background:**

Classical mammalian mitogen-activated protein kinase (MAPK) pathways consist of a cascade of three successive phosphorylation events resulting in the phosphorylation of a variety of substrates, including another class of protein kinases referred to as MAPK-activating protein kinases (MAPKAPKs). The MAPKAPKs MK2, MK3 and MK5 are closely related, but MK2 and MK3 are the major downstream targets of the p38^MAPK ^pathway, while MK5 can be activated by the atypical MAPK ERK3 and ERK4, protein kinase A (PKA), and maybe p38^MAPK^. MK2, MK3, and MK5 can phosphorylate the common substrate small heat shock protein 27 (HSP27), a modification that regulates the role of HSP27 in actin polymerization. Both stress and cAMP elevating stimuli can cause F-actin remodeling, but whereas the *in vivo *role of p38^MAPK^-MK2 in stress-triggered HSP27 phosphorylation and actin reorganization is well established, it is not known whether MK2 is involved in cAMP/PKA-induced F-actin rearrangements. On the other hand, MK5 can phosphorylate HSP27 and cause cytoskeletal changes in a cAMP/PKA-dependent manner, but its role as HSP27 kinase in stress-induced F-actin remodeling is disputed. Therefore, we wanted to investigate the implication of MK2 and MK5 in stress- and PKA-induced HSP27 phosphorylation.

**Results:**

Using HEK293 cells, we show that MK2, MK3, and MK5 are expressed in these cells, but MK3 protein levels are very moderate. Stress- and cAMP-elevating stimuli, as well as ectopic expression of active MKK6 plus p38^MAPK ^or the catalytic subunit of PKA trigger HSP27 phosphorylation, and specific inhibitors of p38^MAPK ^and PKA prevent this phosphorylation. Depletion of MK2, but not MK3 and MK5 diminished stress-induced HSP27 phosphorylation, while only knockdown of MK5 reduced PKA-induced phosphoHSP27 levels. Stimulation of the p38^MAPK^, but not the PKA pathway, caused activation of MK2.

**Conclusion:**

Our results suggest that in HEK293 cells MK2 is the HSP27 kinase engaged in stress-induced, but not cAMP-induced phosphorylation of HSP27, while MK5 seems to be the sole MK to mediate HSP27 phosphorylation in response to stimulation of the PKA pathway. Thus, despite the same substrate specificity towards HSP27, MK2 and MK5 are implicated in different signaling pathways causing actin reorganization.

## Background

The mitogen-activated protein kinase (MAPK) pathways control crucial cellular processes such as proliferation, differentiation, cell survival, apoptosis, gene regulation, and motility [1,2]. The typical mammalian MAPK pathways include the well-characterized MEK1-2/ERK1-2, JNK1-3, MEK5/ERK5, and p38^MAPK ^pathways, while the atypical MAPK pathways include the less studied ERK3, ERK4, and ERK7 [2-6]. The classical MAPK pathways consist of a three partite module in which a MAPK kinase kinase phosphorylates and activates downstream MAPK kinases, which in turn phosphorylate and activate MAPK. MAPK can then phosphorylate non-protein kinase substrates or yet other protein kinases. The latter are referred to as mitogen-activated protein kinase-activating protein kinases (MAPKAPK) [1,2]. Based on structural and functional homology, the MAPKAPKs are divided in four subfamilies: ribosomal S6 kinase (RSK) with the members RSK1-4; mitogen- and stress-activated kinase (MSK) comprising MSK1 and MSK2; MAPK-interacting protein kinase (MNK) including MNK1 and MNK2, and the MAPK-activated protein kinases (MAPKAPK) MK2, MK3 and MK5 [2-6]. Activation of the MEK1-2/ERK1-2 pathway is predominantly triggered by growth factors and can lead to activation of the RSKs and MSKs. The p38^MAPK ^pathway, which is induced by cellular stress stimuli and cytokines, can activate MNK1/2, MSK1/2 and MK2, 3 and 5 [4-8]. MK5 is also a genuine substrate for the atypical MAPK ERK3 and ERK4, but the stimuli triggering ERK3 and ERK4 are unknown [9].

MK2, MK3 and MK5 are closely related, but MK2 and MK3 are more identical to each other than to MK5 [6]. Cell culture experiments and studies with MK2, MK3, and MK2/MK3 deficient mice have shown that MK2 and MK3 share physiological functions, including cytokine production, endocytosis, architecture of the cytoskeleton, cell migration, cell cycle control, chromatin remodeling, and gene expression [10]. MK5^-/- ^mice on a mixed 129 × C57BL/6 genetic background appear normal, but are more susceptible to chemically-induced skin cancer [11,12], while MK5 deficient mice on a C57BL/6 genetic background display embryonic lethality [13]. Despite their obvious distinct functions, MK2, MK3, and MK5 share at least one known common substrate, HSP27, which they can phosphorylate at serine residues 15, 78, and 82 *in vitro *and *in vivo *[14]. However, the expression levels and activity of these three kinases varies in different cell types, with MK2 more abundantly expressed than MK3 in all cells examined [10]. The relative concentrations of MK2 and MK5 have not been systematically examined, but we found that the relative MK2 protein levels are higher than those of MK5 in HEK293 and HeLa cells (our unpublished results). MK2 seems to be the primary target of p38^MAPK ^[6,15-17], and it is generally accepted that MK2 is the major HSP27 kinase that phosphorylates HSP27 and induces F-actin rearrangement in response to cellular stress because overexpression of a dominant negative MK2, depletion of MK2 by siRNA, MK2 inhibitors, or knockout of MK2 abrogated stress-induced HSP27 phosphorylation [11,18-23]. We have previously shown that activation of the cAMP/PKA signaling pathway triggers phosphorylation, activation, and nuclear export of MK5 [24]. Furthermore we showed that a constitutive active MK5 mutant that resides exclusively in the cytoplasm can phosphorylate HSP27 at the relevant phosphoacceptor sites Ser-78 and Ser-82 *in vivo *and is sufficient to cause reorganization of the cytoskeleton [24,25]. MK2 is not activated by PKA and is not excluded from the nucleus after activation of the cAMP/PKA pathway [24,25], suggesting that the PKA pathway may utilize MK5 to induce phosphorylation of HSP27. Here we demonstrate that stress-induced phosphorylation of HSP27 is mediated by p38^MAPK^-MK2, while MK5, but not MK2, is implicated in PKA-triggered HSP27 phosphorylation. These results indicate that although MK2 and MK5 may play a role in a common cellular process such as F-actin reorganization, they operate in different pathways to do so.

## Materials and methods

### Materials

We used the adenylate cyclase activator forskolin from both Enzo Life Sciences (Plymouth Meeting, PA, USA) and from Sigma-Aldrich (St. Louis, MO, USA), while the p38^MAPK ^inhibitor SB203580 was purchased from Enzo Life Sciences. The PKA inhibitor H89 was from Bioaffin GmbH&Co KK (Kassel, Germany). Sodium arsenite was obtained from Sigma-Aldrich. Recombinant MK2, MK3 and MK5 were purchased from Invitrogen Corporation (Carlsbad, CA, USA), while purified Recombinant HSP27 with a C-terminal six histidine tag was obtained from R&D Systems (Minneapolis, MN, USA; cat. no. 1580-HS). Purified HSP27 with an N-terminal GST tag was from SignalChem (Richmond, Canada). Anti-HSP27 antibodies were purchased from Millipore (Billerica, MA, USA; cat. no. MAB88051) and Santa Cruz Biotechnology (Santa Cruz, CA, USA; cat. no. sc-9012). MK2 and MK3 antibodies were from Cell Signaling Technology (Danvers, MA, USA; cat. no. 3042 and 3043, respectively), while PRAK antibodies were from Santa Cruz (cat. no. sc-46667). Phosphospecific antibodies against phosphorylated HSP27 at serine 78, and 82 were purchased from Millipore (cat. no. 04-447 and 04-448), Epitomics (cat. no. 1543-1 and 1118-1), and Cell Signaling Technology (cat. no. 2405 and 2401). PhosphoSer-15 Hsp27 antibody was from Cell Signaling technology (cat. no. 2404) and Upstate Biotechnology (Lake Placid, NY, USA; cat. no. 07-388). The phosphoMK2 Thr222 and Thr334 were both from Cell Signaling Technology (cat. no. 3316 and 3007, respectively). Expression plasmids for the catalytic subunit Cα of PKA, EGFP-MK5, Flag-tagged HSP27, Flag-tagged HSP27-3A, MKK6, and p38^MAPK ^have been previously described [24,29,31].

### Cells

HEK293 cells (ATCC CRL-1573) were purchased from the American Type Culture Collection (LGC Standards, Boras, Sweden) and maintained in Eagle's minimum essential medium supplemented with 10% fetal calf serum, 2 mM L-glutamine, penicillin (110 units/ml), and streptomycin (100 μg/ml).

### In vitro kinase assay

Phosphorylation of recombinant HSP27 by MK2, MK3, and MK5 was performed in 25 mM Tris.HCl, pH 7.5; 10 mM MgCl_2_; 0.05 mg/ml BSA; 2.5 mM dithiothreitol; and 0.15 mM cold ATP in a total volume of 40 μl at 30°C for 30 min. The reactions were stopped in 4xLDS sample buffer and the proteins were denaturated at 70°C for 10 min. The samples were then analyzed by polyacrylamide gel electrophoresis (PAGE) and phosphorylation was monitored by western blotting using phospospecific antibodies (see below).

### Transfections

The day before transfection, 3×10^5 ^cells were seeded out per well in a six-well plate. The next day, cells were transfected with Lipofectamine 2000 (Invitrogen) according to the manufacturer's instructions. Scrambled and siRNA directed against MK2, MK3 and MK5 were purchased from Ambion, Applied Biosystems (Foster City, CA, USA).

### Western blotting

For detection of specific (phospho)proteins, samples were analyzed by ClearPAGE 4-12% BisTris SDS-PAGE (C.B.S. Scientific Company, Solana Beach, CA, USA) or NuPage (Invitrogen Life Technologies) according to the manufacturer's protocol and blotted onto a 0.45-μm polyvinylidene difluoride membrane (Millipore, Billerica, MA). Incubation of the membrane with antibodies was performed as previously described [24]. Briefly, the membrane was blocked with PBS-T (PBS with 0.1% Tween 20 (Sigma) containing 10% (w/v) dried skimmed milk for 1 h and subsequently probed overnight at 4°C with the primary antibody of choice. The next day, the membrane was washed three times with PBS (or PBS-T for the phospho-specific antibodies) and incubated with the appropriate secondary antibody for 1 h at room temperature. Visualization of proteins was achieved by using CDPStar substrate (Tropix, Bedford, MA) and Lumi-Imager F1 from Roche Applied Science. MagicMark™ western standard from Invitrogen Life Technologies was used to estimate the molecular mass of the detected proteins.

### RNA isolation and reverse transcriptase polymerase chain reaction (RT-PCR)

RNA was isolated from HEK293 cells using total RNA isolation NucleoSpin^® ^RNA II kit from Macherey-Nagel (Düren, Germany) according to the manufacturer's instructions. The RNA concentration and purity were checked by spectrometry using a Nanodrop ND-1000 (NanoDrop Technologies Inc., Wilmington, DE, USA). Two μg of RNA was reversed transcribed using the iScript cDNA synthesis kit (BioRad, Biocompare, San Francisco, CA, USA) and subjected to PCR. To amplify MK2, MK3, and MK5 cDNA following primers were used: MK2 forward: GAATCTGTACGCAGGGAGGAAG; MK2 reverse: CATACTGGCCCATTCGGAT; MK3 forward GTTGTCCAAGCAGGTGCTGGG, MK3 reverse: TTAAGCACTGCGTCTTTCTCC; MK5 forward: CCCTACACTTACAACAAGAGCTGTG, and MK5 reverse: CTTTATCTGTGAATCCACGACCATTC. The primers for rRNA have been previously described [33]. The PCR conditions were denaturation at 94°C for 30 sec, annealing for 30 sec at 60°C, and extension for 1 min at 72°C. For rRNA, 20 cycles were run, while for MK2, MK3, and MK5, 30 cycles were used. Amplicons of 479 bp (MK2), 420 bp (MK3), 691 bp (MK5) and 345 bp (rRNA) are obtained.

### Densitometry

Densitometry was performed using a BioRad Model GS-700 Imaging Densitometer and the Multi-Analyst version 1.1 Software.

## Results

### MK2, MK3, and MK5 phosphorylate HSP27 in vitro at serine residues 15, 78 and 82 but with different stoichiometry

Previous studies have shown that activation of the p38^MAPK ^pathway induces phosphorylation of HSP27 and that p38^MAPK ^downstream targets MK2, MK3, and MK5, are genuine HSP27 kinases [7,14]. Moreover, we have demonstrated that activation of the cAMP/PKA pathway potentiates the kinase activity of MK5 [24]. The aim of this study was to examine whether activation of the PKA pathway causes phosphorylation of HSP27 and to elucidate the involvement of MK2, MK3, and MK5 in p38^MAPK^- and PKA-induced HSP27 phosphorylation.

**Figure 1:**
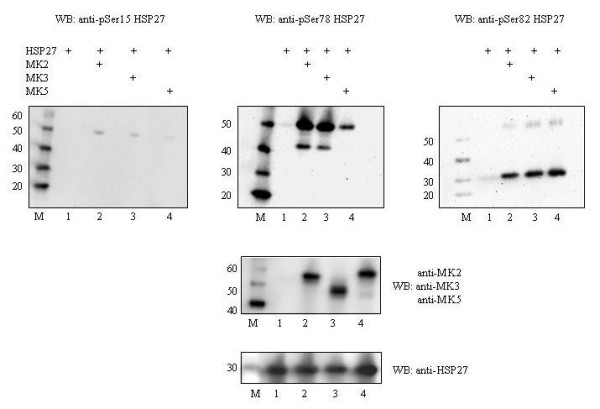
**Detection of phosphoHSP27 by different phosphospecific antibodies after *in vitro *phosphorylation with MK2, MK3, or MK5**. *In vitro *kinase assay was performed with either 200 ng recombinant GST-HSP27 (~52 kD; left and middle top panels) or histidine-tagged HSP27 (~28 kD; right top panel) in the presence of purified MK2 (60 μg), MK3 (96 μg), or MK5 (90 μg). Phosphorylation at the relevant phosphoacceptor sites Ser-15, Ser-78, and Ser-82 was monitored by western blot using phosphospecific antibodies against phosphoSer-15 (Upstate), phosphoSer-78 (Cell Signaling Technology), and phosphoSer-82 (Milipore). Top panel, left: phosphorylation at Ser-15; top panel middle: phosphorylation at Ser-78; top panel right: phosphorylation at Ser-82. The presence of MK2, MK3, or MK5 was verified by western blot with anti-MK2, anti-MK3, and anti-MK5 antibodies (middle panel). Equal loading of HSP27 was monitored with anti-HSP27 antibodies (bottom panel). M = protein molecular mass marker (in kD).

The availability of specific antibodies against phosphoSer-15, phosphoSer-78, and phosphoSer-82 allows rapid and convenient monitoring of HSP27 phosphorylation at these sites in cells. To test the utility of these phosphospecific antibodies, we examined commercially available phosphoSer-15, phosphoSer-78, and phosphoSer-82 antibodies from different commercial sources (see Table 1) to recognize *in vitro *phosphorylated HSP27. Recombinant GST-HSP27 or His-tagged HSP27 were incubated with purified active MK2, MK3, or MK5, respectively, and phosphorylation of Ser-15, -78, and -82 was monitored by phosphospecific antibodies. Increased *in vitro *phosphorylation, although with different stoichiometry, could be detected at Ser-15, Ser-78 and Ser-82 in the presence of MK2, MK3, and MK5 (Figure 1). The phosphorylation by MK5 was less potent than by MK2 and MK3, despite the use of similar amounts of MK2 (60 μg), MK3 (96 μg) and MK5 (90 μg) in our assay. We decided to use the phosphoSer-78 and phosphoSer-82 antibodies for our studies in cells.

**Table 1:** Sources of the commercially available phosphoHSP27 specific antibodies used in this study.

antibody/source	Millipore	Epitomics	CST*	Upstate
phosphoSer-15			+	+
phosphoSer-78	+	+	+	
phosphoSer-82	+	+	+	

*Cellular Signaling Technology

### HEK293 cells express different transcript and protein levels of MK2, MK3 and MK5

**Figure 2:**
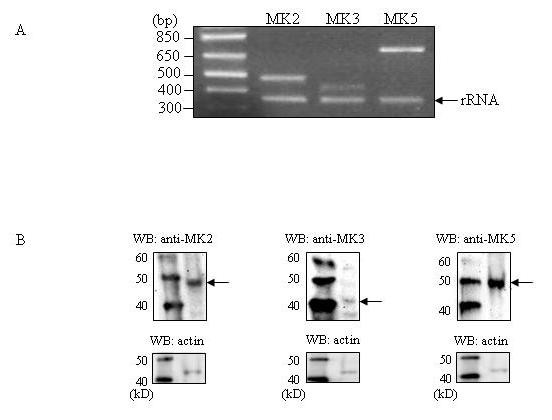
**Expression levels of MK2, MK3, and MK5 in HEK293 cells**. (A) Total RNA was reverse transcribed and MK2, MK3, MK5, and rRNA transcripts were amplified by PCR with specific primers. PCR products were visualized by electrophoresis on an agarose gel in the presence of ethidium bromide. A size marker (in base-pairs; bp) was run on the gel to confirm the correct size of the PCR products. rRNA was used as an internal control [33]. (B) The relative amounts of MK2, MK3, and MK5 in HEK293 cells was determined by western blotting. The band corresponding to the molecular mass of MK2, MK3, and MK5 is indicated by an arrow. To assure equal sample loading, the blot was stripped and re-probed with antibodies against actin. The molecular mass (in kD) of a protein marker is shown.

Because we wanted to perform our studies in HEK293 cells, we examined the expression of MK2, MK3, and MK5 at the RNA and protein level in these cells. Total RNA was reverse transcribed and then amplified with specific primers for MK2, MK3, and MK5. The relative MK3 mRNA expression levels are less than those of MK2 and MK5 (Figure 2A). This is in agreement with a previous study that showed that MK2 transcript levels detected by reverse transcription-PCR (RT-PCR) were more abundant than those of MK3 [10]. Human MK2, MK3, and MK5 have a molecular mass of 47 kD, 42 kD, and 54 kD, respectively [6]. All three kinases were expressed in these cells, but the protein levels of MK3 appeared lower than MK2 and MK5 (Figure 2B), although it can be argued that differences may be due to variable quality of the different antibodies. This is, however, unlikely because another group using MK3 antibodies of a different commercial source made the same observation [10]. Thus, both RT-PCR and western blot assays confirm that MK2 and MK5 are more vividly expressed than MK3.

### Activated PKA pathway can trigger HSP27 phosphorylation

**Figure 3:**
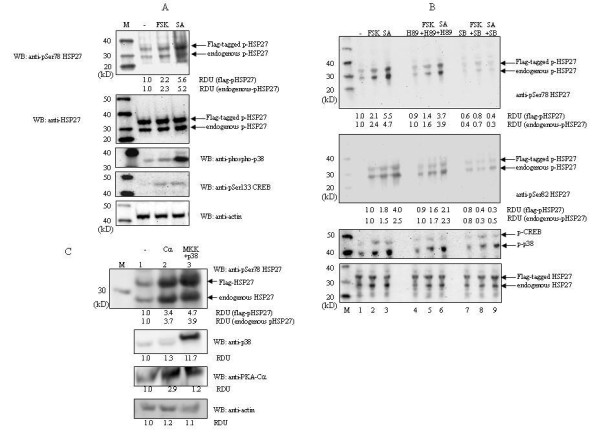
**Activation of the p38^MAPK ^and PKA pathways can provoke phosphorylation of HSP27**. (A) HEK293 cells were transfected with an expression vector for Flag-tagged HSP27 and 24 h after transfection left untreated (-) or exposed to forskolin (FSK; 10 μM for 30 min) or sodium arsenite (SA; 250 μM for 30 min). The phosphorylation levels of phosphoSer-78 HSP27 were monitored. Equal loading was verified by examining the total levels of HSP27 and actin in the samples. To ensure that the stimuli were active, phosphorylation of the PKA substrate CREB at Ser-133 and of the p38^MAPK ^was tested. The double bands observed in the blots with (phospho)HSP27 antibodies represent Flag-tagged HSP27 (upper band) and endogenous HSP27 (lower band). (B) HEK293 cells transfected with the Flag-HSP27 expression plasmid and cells were either left untreated, exposed for 30 min with the PKA inhibitor H89 (10 μM) or the p38^MAPK ^inhibitor SB203580 (10 μM) before forskolin or arsenite was added, or were treated with forskolin or arsenite as described in (A). Lane 1: untreated Flag-Hsp27 transfected HEK293 cells; lane 2: stimulated with forskolin; lane 3: stimulated with sodium arsenite; lane 4: exposed to H89; lane 5: pretreated with H89 and then stimulated with forskolin; lane 6: pretreated with H89 and then stimulated with arsenite; lane 7: exposed to SB203580; lane 8: pretreated with SB203580 and then stimulated with forskolin; lane 9: pretreated with SB203590 and then stimulated with arsenite. PhosphoSer78 HSP27 and phosphoSer82 HSP27 were examined. Membranes were stripped and the lysates were assayed for phosphorylation of CREB and p38^MAPK^. Lower panel shows the expression levels of endogenous and Flag-tagged HSP27. (C) Cells were cotransfected with expression vectors for Flag-tagged HSP27 and the catalytic subunit of PKA (Cα) or p38^MAPK ^and a constitutive active mutant of its upstream activator MKK6 (MKK). PhosphoSer78 HSP27 protein levels were examined by western blotting. M is the protein molecular mass (in kD) marker. RDU (relative densitometry units) indicates the increase in HSP27 phosphorylation and was calculated as follows. The densitometry values obtained for the signals of phosphorylated HSP27 were determined and corrected for the values obtained for actin (in A) or for total HSP27 (in B). This ratio obtained for untreated cells was arbitrarily set as 1.0 and the ratios obtained for stimulated cells were related to this. Values were calculated separately for flag-tagged HSP27 and endogenous HSP27.

To test whether activation of the PKA pathway results in phosphorylation of HSP27, Flag-tagged HSP27 transfected HEK293 cells were treated with the cAMP elevating agent forskolin and phosphorylation of HSP27 at Ser-78 and Ser-82 was monitored by western blot using phosphoSer-78 and phosphoSer-82 specific antibodies. Forskolin treatment resulted in >2-fold increase in HSP27 phosphorylation at Ser-78 and Ser-82 (Figure 3A and 3B). Activation of the p38^MAPK ^pathway by sodium arsenite triggered HSP27 phosphorylation at both sites (Figure 3A and 3B). Forskolin (respectively arsenite) treatment enhanced phosphorylation of the PKA substrate CREB (respectively phosphorylation of p38^MAPK^), indicating that the stimuli were functional (Figure 3A). In agreement with previous studies, arsenite also caused CREB phosphorylation, probably through MSK1 [4,8]. Pretreatment of the cells with the PKA specific inhibitor H89 reduced forskolin-induced HSP27 phosphorylation, indicating the involvement of PKA (Figure 3B). The specific p38^MAPK ^inhibitor SB203580 almost completely abrogated arsenite-induced HSP27 phosphorylation (Figure 3B). To provide further experimental proof that PKA can mediate phosphorylation of HSP27, we cotransfected cells with expression plasmids for the catalytic subunit of PKA (Cα) and Flag-tagged HSP27. Increased phosphorylation of endogenous and Flag-tagged HSP27 was observed in cells ectopically expressing PKA-Cα compared to cells transfected with empty vector (compare lanes 1 and 2 in Figure 3C).

### MK2 and MK5 contribute differently to stress- and PKA-induced HSP27 phosphorylation

**Figure 4:**
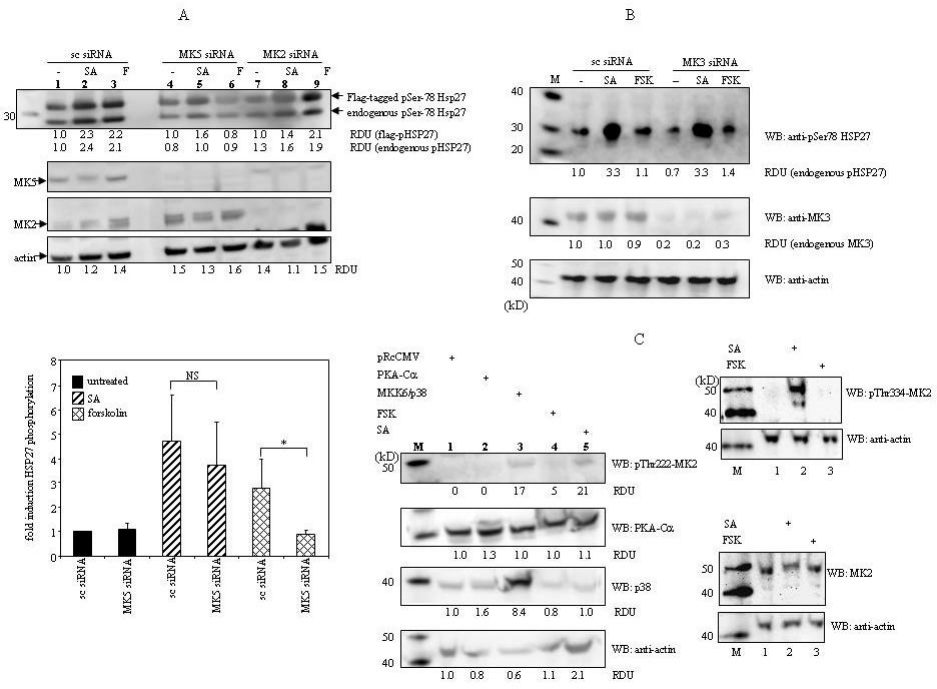
**MK5 is implicated in PKA-induced HSP27 phosphorylation, while MK2 mediated p38^MAPK^-triggered HSP27 phosphorylation**. (A) Top panel: cells were transfected with scrambled siRNA (sc siRNA; lanes 2-4) or siRNA directed against MK5 (MK5 siRNA, lanes 4-6) or MK2 (lanes 7-9). After transfection, cells were left untreated (-, lanes 1, 4 and 7) or treated for 30 min with 250 μM sodium arsenite (SA, lanes 2, 5 and 8) or 10 μM forskolin (FSK, lanes 3, 6 and 9) and the cells were harvest. Protein levels were examined by western blot. Equal loading was verified by examining the expression levels of actin. M = protein molecular mass marker (in kD). The intensity of the bands was determined by densitometry and the value obtained for phosphorylated Flag-tagged-HSP27 (respectively phosphorylated endogenous HSP27) was arbitrary set as 1.0 and the other values were related to this. A representative experiment is shown and similar results were obtained in two independent experiments. Bottom panel: relative fold induction of HSP27 phosphorylation at Ser-78 in untreated and treated cells that had been transfected with scrambled (sc) or MK5-targeting siRNA. The phosphoHSP27 levels in untreated, scrambled siRNA transfected cells were arbitrary set as 1.0. The bars represent the average (+standard deviation) of three independent results. A significant difference (*p < 0.05; student's *t*-test) was observed for forskolin-induced HSP27 phosphorylation in scrambled siRNA and MK5-targeting siRNA transfected cells. (B) As in (A), but cells were transfected with siRNA against MK3. RDU values were calculated as in (A). (C) Overexpression of active MKK6 plus p38^MAPK ^(lane 3) or exposure of cells to 250 μM sodium arsenite (SA) for 30 min (lane 5) causes phosphorylation of MK2, while the catalytic subunit of PKA (Cα) or forskolin (FSK) failed to provoke HSP27 phosphorylation. Control cells were transfected with the empty expression vector pRcCMV (lane 1). The molecular mass (in kD) of the protein marker is shown (lane M). The densitometry values obtained for the signals of phosphorylated HSP27 or MK5 (respectively MK2 or MK3) were determined and corrected for the values obtained for actin. RDU values in control cells (lanes 1) were arbitrarily set as 1.0 and the other values were related to this.

Our results suggest that PKA can mediate phosphorylation of HSP27 in cells, but the putative role of MK2, MK3, and MK5 has not been addressed. On the other hand, it is generally accepted that cellular stress triggers HSP27 phosphorylation through the p38^MAPK^/MK2 signaling pathway [26]. However, p38^MAPK ^can also activate MK3 and MK5 [7], suggesting that these protein kinases may also be implicated in stress-induced HSP27 phosphorylation. This prompted us to scrutinize a possible involvement of MK2, MK3, and MK5 in stress- and PKA-induced HSP27 phosphorylation. The implication of the MKs 2, 3 and 5 was tested by examining HSP27 phosphorylation in siRNA-treated cells. Transfection of HEK293 cells with scrambled siRNA had no effect on the phosphoHSP27 levels after arsenite or forskolin treatment (Figure 4A). Depletion of MK5 diminished forskolin triggered HSP27 phosphorylation, but not arsenite. Knockdown of MK2 strongly reduced arsenite-triggered HSP27 phosphorylation, but did not affect HSP27 phosphorylation caused by forskolin (Figure 4A). Our results demonstrated no obvious effect on arsenite- and forskolin-induced HSP27 phosphorylation in cells with knockdown expression of MK3 (Figure 4B). An additional experimental approach was used to explore the possible role of MK2 in arsenite and forskolin-induced HSP27 phosphorylation. While sodium arsenite clearly triggered MK2 activation as monitored by phosphorylation of Thr-222 and Thr-334 [6,10], no MK2 phosphorylation was observed in cells exposed to forskolin (compare lanes 4 and 5 in the left panel of Figure 4C and lanes 2 and 3 in the middle panel of Figure 4C). Similarly, MK2 phosphorylation was detected in cells transfected with expression plasmids for active MKK6 plus p38^MAPK^, but not in cells transfected with the catalytic subunit of PKA (compare lanes 2 and 3 in the left panel of Figure 4C). Hence, these data indicate that sodium arsenite, but not forskolin can activate the HSP27 kinase MK2.

Taken together, these results indicate that MK2 is the major HSP27 kinase in stress-induced cells, while MK5 mediates HSP27 phosphorylation upon activation of the PKA pathway.

## Discussion

HSP27 is a multifunctional protein and some of its functions are regulated by phosphorylation mediated by different pathways and protein kinases [14]. The MAPKAPK MK2, MK3, and MK5 can all phosphorylate HSP27 *in vitro *and *in vivo *[14] and are downstream targets of p38^MAPK ^[6,7], while MK5 can also be activated by PKA [24,25]. However, the implication of MK5 in p38^MAPK^- and PKA-induced HSP27 phosphorylation remains incompletely explored. The aim of this study was therefore to investigate the contribution of MK2, MK3, and MK5 in p38^MAPK^- and PKA-trigger HSP27 phosphorylation.

### MK3 is not a major HSP27 kinase in p38^MAPK ^and PKA-induced HSP27 phosphorylation

Although HSP27 was reported to be a good MK3 substrate, at least *in vitro *[27], no HSP27 phosphorylation was detected in MK2^-/- ^mouse embryonic fibroblasts (MEF) after p38^MAPK ^activation [11]. This indicates that MK3 does not detectably contribute to stress-induced phosphorylation of HSP27 in MEF cells. In agreement with the findings of Shi and his colleagues, we found that depletion of MK3 did not affect HSP27 phosphorylation levels in HEK293 cells with activated p38^MAPK ^or PKA signaling pathways. The group of Gaestel found lower expression levels and activity of MK3 compared to MK2 in all cells tested [10]. We also observed much lower MK3 transcript and protein levels than MK2 and MK5 in HEK293 cells (Figure 2). The low protein levels of MK3 may therefore explain the inferior or ignorable activity of MK3 towards HSP27. We can, however, not exclude a role for MK3 as a HSP27 kinase in other pathways or other cells.

### MK5, but not MK2 is implicated in PKA-induced HSP27 phosphorylation

Both MK2 and MK5 phosphorylated HSP27 at Ser-78 and Ser-82 *in vitro*, although with different stoichiometry (Figure 1). While MK2 could also phosphorylate Ser-15, MK5 had only weak kinase activity towards this site. This is in agreement with our previous study where we failed to detect *in vivo *phosphorylation of this residue by MK5 [25]. The *in vitro *results indicate that HSP27 may be a better substrate for MK2 or that the intrinsic kinase activity of MK2 is higher than MK5. In agreement with this assumption, the group of Matthias Gaestel reported that immunoprecipitated MK5 from arsenite-treated MEF cells had a 6-times lower activity towards Praktide than MK2 immunoprecipitated from arsenite-treated cells [11]. Our study confirms the dominant role of MK2 in stress-induced phosphorylation of HSP27, while MK5 does not seem to contribute in this process. On the other hand, MK5, but not MK2 seems to operate as the HSP27 kinase in response to activation of the cAMP/PKA pathway. Recently, we reported that PKA phosphorylates and activates MK5 *in vitro *[24], while we and others had shown that MK5 can phosphorylate HSP27 *in vivo *[28,29]. However, our previous results demonstrated that PKA enhanced the kinase activity of MK5 only 2-fold [24]. The lower intrinsic kinase activity of MK5 compared to MK2 and the weak induction by PKA (2-fold) may explain why forskolin or overexpression of the catalytic subunit of PKA only triggered a moderate increase in HSP27 phosphorylation compared to arsenite or active MKK6/p38^MAPK^.

MK2 does not participate in PKA-induced HSP27 phosphorylation in HEK293 cells, an assumption that is underscored by our previous finding that PKA did not phosphorylate MK2 [24]. Phosphorylation of MK5 at Thr-182 by the p38^MAPK ^pathway has been shown to be necessary for activation *in vitro *[30,31], but the *in vivo *interaction between p38^MAPK ^and MK5 remains controversial [7]. The role of MK5 as a genuine HSP27 kinase in stress-treated cells has been jeopardized by the studies of Shi and co-workers [11]. These authors failed to detect phosphoHSP27 in arsenite-treated MK2^-/- ^MEF cells, but not in MK5^-/- ^MEFs. Moreover, they were unable to phosphorylate recombinant HSP27 with immunoprecipitated MK5. Our siRNA studies confirmed that MK5 is not involved in arsenite-induced HSP27 phosphorylation (Figure 4A). The obvious explanation why MK5 does not participate in p38^MAPK^-induced HSP27 phosphorylation is that MK5 is not a *bona fide *substrate for p38^MAPK ^[7].

Our results demonstrate that MK5 is required, whereas MK2 and MK3 appear dispensable for PKA-induced HSP27 phosphorylation. The selectivity of PKA for MK5 can be explained as follows. We have recently demonstrated that Ser-115 is an *in vitro *PKA phosphoacceptor site and that ectopic expression of the phosphomimicking MK5 S115D mutant resulted in potent *in vivo *phosphorylation of HSP27, while the MK5 S115A mutant was unable to do so [25]. Interestingly, the corresponding Ser-115 residue is not conserved in MK2 (and MK3) and may explain why PKA cannot activate MK2. Moreover we have shown that MK5 and the catalytic Cα subunit of PKA can form complexes in cells, while MK2 and Cα subunit cannot be immunoprecipitated [24]. Hence MK5, but not MK2 and MK3 may represent a substrate for PKA.

Both stress and cAMP elevating signals can cause F-actin rearrangements [32]. Phosphorylation of HSP27 allows oligmerization of F-actin and cytoskeleton remodeling. The involvement of the p38^MAPK^-MK2-HSP27 pathway in F-actin rearrangements is well illustrated [6]. The PKA-MK5-HSP27 connection unveiled in this study and our previous findings that active MK5 can induce F-actin rearrangement in a HSP27-dependent manner [29] may explain one mechanism for PKA-induced F-actin reorganization.

We also showed that both the p38^MAPK ^and the PKA pathway can cause HSP27 phosphorylation at Ser-78 and Ser-82. We did not test phosphorylation at Ser-15 because the antibodies we used gave weak signals for any of the three kinases using *in vitro *phosphorylated HSP27. We were previously unable to detect HSP27 phosphorylation at Ser-15 in cells transfected with an active MK5 mutant, although increased phosphorylation on Ser-78 and Ser-82 was observed [29]. At present we do not know whether MK5 can phosphorylate HSP27 at Ser-15 *in vivo *or whether the failure to detect phosphorylation was the result of poor antibody quality.

## Conclusions

**Figure 5:**
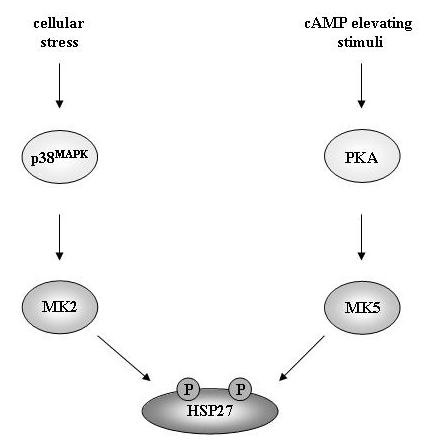
**Different stimuli that trigger HSP27 phosphorylation engage distinct pathways with specific MKs**. MK2 mediates phosphorylation of HSP27 in cells with an activated p38^MAPK ^pathway, while MK5 functions as an HSP27 kinase induced by the cAMP/PKA pathway.

In conclusion, we suggest that both MK2 and MK5 are genuine HSP27 kinases that are engaged in different pathways. While MK2 mediates phosphorylation of HSP27 in response to stress-induced activation of the p38^MAPK ^pathway, MK5 seems to be involved in phosphorylation of HSP27 triggered by PKA (Figure 5). Aberrant HSP27 phosphorylation levels have been observed during viral infections, in diabetic kidney and heart, and in diseases such as cancer, the autoimmune diseases pemphigus vulgaris and pemphigus foliaceus, and kidney fibrosis [14]. Hence, meticulous mapping of the different pathways and protein kinases involved in perturbed HSP27 phosphorylation in these clinical conditions may allow the design of specific therapeutic approaches to prevent abnormal phosphoHSP27 levels.

## List of Abbreviations

HSP27: heat shock protein 27; PKA: cAMP-dependent protein kinase or protein kinase A; MAPK: mitogen-activated protein kinase; MAPKAPK: MAPK-activated protein kinase; MK: MAPKAPK;

## Competing interests

The authors declare that they have no competing interests.

## Authors' contributions

AS, GD, and UM designed the experiments, interpreted and discussed the results. AS, GD and UM performed the experiments. UM wrote the first draft of the manuscript. AS and GD contributed with writing the manuscript. All authors read and approved the final manuscript.
